# Personal inhalable paper dust exposure and potential determinants among paper industry workers in Ethiopia

**DOI:** 10.1007/s00420-024-02097-5

**Published:** 2024-08-13

**Authors:** Ararso Tafese, Abera Kumie, Teferi Abegaz, Samson Wakuma Abaya, Bente E. Moen, Wakgari Deressa, Magne Bråtveit

**Affiliations:** 1 Department of Preventive Medicine, School of Public Health, Addis Ababa University, P.O. Box 90861000, Addis Ababa, Ethiopia; 2 Occupational and Environmental Medicine, Department of Global Public Health and Primary Care, University of Bergen, Bergen, 5020 Norway; 3 Centre for International Health, Department of Global Public Health and Primary Care, University of Bergen, Bergen, 5009 Norway

**Keywords:** Exposure assessment, Exposure model, Paper dust, Paper industry, Occupational exposure

## Abstract

**Purpose:**

Excessive paper dust during paper production may harm the workers’ respiratory health. We wanted to assess the inhalable paper dust levels and its determinants among paper industry workers.

**Methods:**

A study was conducted in Ethiopia to assess the level of personal inhalable paper dust exposure among four paper mills. A total of 150 samples were collected using the IOM sampler attached to Side Kick Casella pumps at a flow rate of 2 L/min. The samples were analyzed in Nemko Norlab, Norway. Linear mixed-effect models were applied to identify determinants of inhalable paper dust.

**Results:**

The geometric mean of personal inhalable paper dust was 3.3 mg/m^3^ with 80% of the measurements exceeding the Swedish occupational exposure limit (OEL) of 2 mg/m^3^. The linear mixed-effects model showed that the level of dust was 28% higher when using high-speed than when using low-speed rewinding machines, while paper mills with an average of more than four machines per job group had 22% higher exposure than paper mills with a lower number of machines. Furthermore, working in packing and preparation was associated with higher dust exposure than in other areas.

**Conclusions:**

The dust exposure levels were above the Swedish OEL for 80% of the samples. This indicates that preventive measures should be established in the industry. The exposure model identified high-speed rewinding machines, a high number of machines, and work in preparation and packing as associated with high levels of paper dust exposure.

## Introduction

Worldwide the production of paper and paper board has been stable over the last decade but was expected to increase over the next ten years for the application of packaging purposes (Bajpai 2015; De Matteis et al. 2017; Martin and Haggith 2018). Wood preparation, pulping, chemical recovery, bleaching, and paper production are all part of the production process of the pulp and paper industry. While wood remains the primary raw material, recycled paper constitutes approximately 50% of the materials used. However in some paper mills cellulose-bearing materials such as straw, hemp, hay, and cotton may be used in the production process (Bajpai 2015; Zhong et al. 2018). These materials are used to manufacture an extensive range of paper and paper products, such as white bond paper, fluting paper, test liners, kraft liners, ream wrappers, manila board, core-making paper, grey board, paper tubes and cones, packets, labels, various types of cardboard, and corrugated carton boxes. Additionally, soft tissue mills employ virgin pulp and high-quality fibers (Alireza 2006) to produce hygienic products like, toilet paper, napkins, paper towels, and facial tissue. Because of the different processes and products, the soft tissue mills might emit different levels of dust than other paper mills.

Several studies in Europe, especially in Sweden, have measured personal inhalable and personal total dust in the soft tissue paper industry (Andersson et al. 2020; Hellgren et al. 2001; Kraus et al. 2002; Neitzel et al. 2022; Torén et al. 1994, 2020; Westberg et al. 2016). Neitzel et al., (Neitzel et al. 2022) reported an overall decline in total dust exposure in four Swedish soft tissue paper mills from 7.0 mg/m^3^ in the 1980s to 1.0 mg/m^3^ in 2009. Dust exposure varied with working sections (Torén et al. 1996), work locations, ventilation, and machines used in papermaking (Neitzel et al. 2022). In a study of Germany’s soft tissue industry (Kraus et al. 2002) the personal inhalable paper dust exposure was reported to be higher, with a mean concentration of 10.3 mg/m^3^ (range 0.2–96.1 mg/m^3^). In 2016 personal inhalable paper dust in Swedish pulp and paper mills was even lower with a mean of 0.30 mg/m^3^ (0.005–3.3 mg/m^3^) (Westberg et al. 2016). The decline in dust exposure over time in Sweden has partly been explained by improved ventilation and dust controls as well as more enclosure of dust-emitting processes (Neitzel et al. 2022). Studies in Sweden have reported that occupational exposure to personal paper dust greater than 5 mg/m^3^ resulted in lung function reduction and increased mortality due to asthma (Andersson et al. 2020; Torén et al. 2020).

However, the results of the European studies may not apply to the paper manufacturing industry in Ethiopia due to various reasons such as differences in workers’ health and safety practices, technologies used in paper-making, and implementation of engineering control mechanisms to reduce paper dust emissions. To our knowledge, only one study has been conducted in Ethiopia to measure dust exposure levels among workers in the paper mill industry. Results from personal measurements of unspecific, total dust fraction using closed-face 37 mm filter cassettes showed an arithmetic mean of 11.3 mg/m^3^ (Negash et al. 2023). However, a larger study using the IOM sampling head for sampling of inhalable particles according to the ISO standard is needed to obtain representative estimates of dust exposure in Ethiopian paper mills. Furthermore, the previous study did not address potential determinants that may have an impact on dust exposure levels and thus may indicate targets for interventions to reduce exposure. Therefore, this study aims to determine the level of personal inhalable paper dust exposure and to identify determinants of paper dust among paper mill industry workers in Ethiopia.

## Methods and materials

Samples of personal inhalable dust were collected from four paper mills in Ethiopia from February 2023 to April 2023. We selected the mills depending on their size (large-scale industries) and the type of raw materials used. These four paper mills are categorized under large-scale industries depending on their human resources of more than 250 employees per industry (Eurostat 2010). Two of the paper mills used both recycled paper and imported pulp as raw materials while the two others used only recycled paper for the production of paper and paper products.

### The paper making process

In the following job groups, various activities were carried out using a combination of manual and machine methods. Each activity was conducted in a designated room. There were no control rooms for the workers in any of the four mills.

#### Preparation

The paper-making starts with the selection of raw materials, typically recycled paper and pulp sourced from various locations. The recycled materials are manually and mechanically segregated from waste materials and poured into a conveyor and bell machine, then subjected to pulping methods to remove impurities and break down fibers, resulting in the formation of pulp. Depending on the desired quality and characteristics of the final product, pulp is further processed through bleaching methods to remove color and improve brightness.

#### Paper machine operation

The pulp slurry is then fed onto a continuous wire mesh conveyor, where water drains away and fibers begin to bond, forming a continuous sheet. The sheet is then passed through rollers to further remove water and compress the fibers, increasing density. The sheet is then dried through a series of heated rollers or drying cylinders and may undergo sizing treatment for improved water resistance or printability.

#### Finishing and converting

During the finishing process, raw paper is transformed into its final product through a series of treatments, including cutting, trimming, coating, laminating, embossing, printing, and more. Paper rolls are then converted into a variety of end products, such as packaging materials, through additional processes.

#### Packing

The packing comes at the end of the paper production process, just before the paper products are shipped out. During this stage, the paper products - whether rolls or sheets of paper - are inspected for quality and then packaged per the customer’s specific requirements or industry standards. The products are protected during transportation and storage with packaging materials such as wrappers, labels, or containers.

### Dust sampling strategies

We defined four job groups of workers based on similarity in the main tasks they performed; Preparation, paper machine, packing, and finishing & converting. According to Rappaport and Kupper (Rappaport and Kupper 2008) the number of personal dust samples per group should be 10 to 20 (2 measurements from each of 5 to 10 workers). Consequently, in each of the four paper mills in our study, five workers were randomly chosen from each group for repeated sampling. A total of 80 workers took part in the study, with each worker being sampled for two consecutive days.

### Measurement of inhalable paper dust

IOM sampling heads attached to Side Kick Casella pumps with a flow rate of 2 L/min were used to collect inhalable personal dust in workers’ breathing zones (OSHA 2014; Skaugset et al. 2013). The pumps were paused during lunch breaks. Full-shift dust measurement was taken on randomly selected days of the week and repeated sampling was conducted on the consecutive day. Data collection took 4–5 days in each paper mill. Calibration was conducted before sampling, and the pumps were checked every hour for the correct flow rate. After the field sampling was completed, the cassettes were transported on hand luggage by airplane to the laboratory. The dust samples were analyzed gravimetrically using a standard microbalance scale AT261 Mettler Toledo with a detection limit of 0.01 mg/m^3^ in the accredited laboratory Nemko, Norlab, Norway. The results were compared with the Swedish occupational exposure limit (OEL) for inhalable paper dust of 2 mg/m^3^ (Swedish Work Environmental Authority 2018). The field blank was not used for analysis purposes due to cross-contamination during the data collection period.

### Descriptions of determinants of paper dust exposure

During the sampling periods, the principal investigator utilized an observational checklist to gather data on potential factors that could contribute to dust exposure. The checklist focused on process technical factors within the job groups, such as the speed of paper rewinding machines which was categorized depending on the mean average above or below 1900 m/minute (Järvinen et al. 2009). The number of machines primarily emitting paper dust in each job group including paper rewinding and slitter machines, baler machines, dryer, and conveyor machines, was counted. The average number of machines in the four job groups was estimated and categorized as greater or less than four machines. Additionally, the feeding of raw materials into the paper mill was categorized as either mechanical or manual. Mechanical ventilation systems were classified as either functioning or non-functioning. A system was considered functioning if it operated effectively by providing the desired airflow, and air exchange and if there was no visible blockages hindering the fan’s operation. The status of housekeeping was categorized as good maintenance when the work areas were maintained in a neat and orderly manner, keeping halls and floors free of slip and trip hazards, and properly disposing of waste materials such as paper and cardboard (Canadian Centre for Occupational Health and Safety 2018). Cleaning of surfaces in the four mills was done manually by sweeping brooms, without any use of vacuum cleaners or pressurized air. The raw materials used in the paper mill were categorized as either recycled paper or a combination of recycled and pulp. We measured the daily air temperature and humidity from all working sections in both mornings (10:00–11:00 AM) and afternoon shifts (2:00–3:00 PM EAT). The mean average indoor temperature and relative humidity were 23.1 °C and 55%, respectively (Table 1).

**Table 1:** Description of potential determinants of personal inhalable paper dust exposure among paper mill industry workers in Ethiopia

Descriptions of potential determinants	Paper mill A	Paper mill B	Paper mill C	Paper mill D
Number of workers	559	270	320	256
Production capacity (tons/day)	64	100	40	30
Year of establishment	1962	2018	1997	2015
Mechanical ventilation	One job group has mechanical ventilation	Two job groups have mechanical ventilation	Two job groups have mechanical ventilation	None
Raw material used	Both recycled & pulp	Both recycled & pulp	Recycled	Recycled
Types of paper and paper products produced	White bond paper, fluting paper, kraft liner, test liner, white kraft liner, ream wrapper, paper tubes and cones, cardboard, corrugated carton boxes	White bond paper, fluting paper, test liner, kraft liner, white top kraft liner, white top test liner, white kraft liner, ream wrapper, manila board, white bristol board, paper tubes and cones,	White bond paper, fluting paper, kraft line, packet, label, cake platter, cone, tube, cardboard, corrugated carton boxes.	Paper, test liner, fluting medium, cardboard, corrugated carton boxes.
Average number of machines in the room (machines distributed in each job group)	6 (4–7)	4 (2–6)	4 (1–9)	2 (1–3)
Speed of rewinding paper machine (m/min)	720	2300	600	2100
Mechanism of feeding of raw materials to the machine	Manual	Mechanical	Manual	Manual
Good housekeeping	Finishing and packing	Paper machine, finishing and packing	Paper machine and finishing	Paper machine, finishing and packing
Daily average indoor air temperature (morning and afternoon) (°C)	24.2 (23.4–25.5)	25.4 (24.7–26.4)	21.47 (19.7– 23.5)	21.0 (20.0–21.5)
Daily average indoor relative humidity (morning & afternoon) (%)	55.9 (54.2–58.8)	53.1 (46.5– 59.3)	51.8 (50.9–52.8)	58.6 (57.7–60.3)

### Data handling and statistical analysis

The distribution of the dust exposure measurements was positively skewed and was log-transformed before analysis. The descriptive results of dust exposure were described by arithmetic mean (AM), geometric mean (GM), and geometric standard deviation (GSD). Independent t-tests were used to test differences in continuous variables between two groups. A one-way ANOVA was used to compare the personal inhalable paper dust exposure levels between job groups. Additionally, a bivariate correlation test was performed to check for any correlation between temperature and log-transformed dust, as well as between relative humidity and log-transformed dust.

Linear mixed effect regression models (SPSS 26) (Abaya et al. 2018; Rappaport and Kupper 2008; van Tongeren et al. 2000) were used to identify significant determinants for personal inhalable paper dust exposure among paper mill industry workers. The log-transformed personal inhalable paper dust exposure level was used as the dependent variable in random and mixed-effect models. In both models, employee and paper mill were entered as random effects. Variables with a significance level of *P* < 0.20 in independent t-tests were entered one by one as fixed effects in a mixed-effects model. The final model contained only determinants with a P-value ≤ 0.05.

The variance component structure model was explained through within-worker (wwδ), between-worker (bwδ), and between-mill (bmδ) variances. The total variance explained by the fixed effects was calculated as the percentage change in the sum of the three variance components between the random and the mixed-effects model. The effects of the significant fixed factors in the mixed models were calculated as e^β^, where β is the regression coefficient.

## Results

### Characteristics of personal inhalable paper dust exposure by paper mill and job groups

Data was collected from 80 workers across four paper mills, resulting in a total of 150 samples. The mean sampling time was 340 min, ranging from 240 to 446 min. The AM and GM for overall personal inhalable paper dust exposure were 4.5 mg/m^3^ and 3.3 mg/m^3^, respectively, with a range of 0.85 to 88 mg/m^3^ (Table 2). Notably, 80% of the samples exceeded the Swedish paper dust OEL of 2 mg/m^3^.

Paper mill B had the highest GM of dust exposure at 4.9 mg/m^3^ with all exceeding the OEL, followed by paper mill D at 3.3 mg/m^3^, while the lowest exposure was found in paper mill C at 2.1 mg/m^3^ (Table 2). The preparation job group of paper mill B had the highest GM of dust exposure at 11.6 mg/m^3^, followed by the finishing and converting job group of paper mill D at 9.2 mg/m^3^. The finishing and converting job group of paper mill C had the lowest dust concentration at 1.5 mg/m^3^.

The concentration of personal inhalable paper dust across different job groups of the workplace is indicated in Table 2. The highest GM value of 4.2 mg/m^3^ was found in the preparation, while the lowest GM value of 2.6 mg/m^3^ was observed in the paper machine job group. According to the one-way ANOVA analysis, there were significant differences in exposure between the 4 job groups within the 4 paper mills as well as between to four groups in the total dataset (Table 2).

**Table 2:** Description of personal inhalable paper dust exposure by paper mill and job groups among paper mill industry workers in Ethiopia

Paper mill	Job groups	Sampling time (min) AM (range)	NW	NS	AM (range) mg/m^3^	GM(GSD) mg/m^3^	% > OEL (2 mg/m^3^)
Paper mill A	Preparation	330 (307–351)	5	10	5.3 (2.6–8.9)	5.0 (1.4)	100%
	Paper machine	316 (283–342)	5	10	2.7 (1.5–4.4)	2.5 (1.5)	70%
	Finishing and converting	361 (317–403)	5	10	3.0 (1.8–4.8)	2.9 (1.4)	90%
	Packing	295 (251–327)	5	10	3.3 (2.5–4.1)	3.3 (1.2)	100%
	Sub-Total	325 (251–403)	20	40	3.6 (1.5–8.9)	3.3 (1.5)***	90%
Paper mill B	Preparation	271 (241–305)	5	10	17.8 (5.6–88.0)	11.6 (2.35)	100%
	Paper machine	264 (240–292)	5	10	3.8 (2.4–5.3)	3.7 (1.3)	100%
	Finishing and converting	390 (361–408)	5	10	3.5(2.2–5.2)	3.4 (1.3)	100%
	Packing	376 (359–400)	5	10	4.0 (2.4–7.6)	3.8 (1.4)	100%
	Sub-Total	325 (240–408)	20	40	7.3 (2.2–88.0)	4.9 (2.0)***	100%
Paper mill C	Preparation	281(241–308)	5	10	2.5 (1.1–4.5)	2.3 (1.6)	50%
	Paper machine	332 (292–355)	5	10	2.0 (1.5–3.6)	1.9 (1.3)	30%
	Finishing and converting	280 (252–325)	5	10	1.7 (0.85–4.1)	1.5 (1.7)	20%
	Packing	335 (260–374)	5	10	3.4 (2.3–5.9)	3.2 (1.4)	100%
	Sub-Total	307 (241–374)	20	40	2.4 (0.85–5.9)	2.1 (1.6)*	50%
Paper mill D	Preparation	432 (397–446)	5	10	2.6 (1.8–5.7)	2.4 (1.4)	60%
	Paper machine	423 (410–445)	5	10	2.5 (1.3–4.1)	2.3 (1.4)	70%
	Finishing and converting	427 (406–437)	5	5	13.4 (3.4–32.0)	9.2 (2.7)	100%
	Packing	413 (391–423)	5	5	6.2 (2.1–15.0)	4.7 (2.2)	100%
	Sub-total	424 (391–446)	20	30	6.2 (1.4–32.0)	3.3 (2.1)**	82.5%
All mills	Preparation	328 (241–446)	20	40	7.1 (1.1–88.0)	4.2 (2.3)	77.5%
	Paper machine	334 (240–445)	20	40	2.7 (1.3–5.3)	2.6 (1.5)	67.5%
	Finishing and converting	355 (252–437)	20	35	4.3 (0.85-32.0)	3.0 (2.1)	74.3%
	Packing	346 (251–423)	20	35	3.9 (2.1–15.0)	3.6 (1.5)	100%
	Grand-Total	340 (240–446)	80	150	4.5 (0.85-88.0) P95 = 10.6	3.3 (1.9)*	80%

OEL: Occupational exposure limit > 2 mg/m^3^, AM = Arithmetic Mean; NS = Number of samples, NW = Number of workers, GM = Geometric mean, GSD = Geometric Standard deviation, P95 = 95th percentile, One-way ANOVA * = *p* < 0.05, ** = *p* < 0.001, *** = *p* < 0.0001

### Potential determinants for paper dust exposure

In unadjusted analysis, there were significant differences in personal exposure to dust within all potential determinants, except for the status of the housekeeping (Table 3). Higher levels of dust were found in paper mills that used more than four machines per job group (3.9 mg/m^3^). Those mills using both recycled and pulp as raw materials also result in higher dust levels when compared to using solely recycled paper. Mills with a production capacity of more than 60 tons/day had higher exposures than mills with less than 40 tons/day. Table 3 shows that the production capacity variable overlaps completely with the raw material variable. The emission of dust was higher when the speed of the rewinding machine was higher (4.1 mg/m^3^) than at the lower speed of the rewinding machine (2.7 mg/m^3^) (Table 3).

The data revealed a significant association between the levels of personal inhalable paper dust and daily temperature (*r* = 0.30, *p* < 0.001), but not between personal inhalable paper dust and relative humidity (*r* = 0.11, *p* = 0.202).

**Table 3:** Potential determinants of log-transformed personal inhalable paper dust exposure among paper mill industry workers in Ethiopia

Determinants	Categories of variables	Personal inhalable paper dust			
		NS	GM (mg/m^3^)	GSD (mg/m^3^)	*p*-value
Functioning of mechanical ventilation	0. Not functioning or absent 1. Functioning	100 50	3.5 2.8	2.0 1.6	0.018
Average number of machines in the room (machines distributed in each job group)	0. ≥ 4 machines 1. < 4 machines	80 70	3.9 2.6	1.8 1.9	< 0.0001
Speed of rewinding paper machines	0. > 1900 m/minute 1. ≤ 1900 m/minute	70 80	4.1 2.7	2.1 1.6	< 0.0001
Raw material used	0. Both pulp & recycled paper 1. Recycled paper	80 70	4.0 2.6	1.8 1.9	< 0.0001
Production capacity	0. > 60 tons/day 1. ≤ 40 tons/day	80 70	4.0 2.6	1.8 1.9	< 0.0001
Mechanism of feeding of raw materials to the machine	0. Mechanical 1. Manual (carrying by hand)	40 110	4.9 2.8	2.0 1.8	< 0.0001
Status of housekeeping by the job group	0. Poor housekeeping 1. Good housekeeping	60 90	3.7 3.0	2.1 1.7	0.075

Independent sample T-tests were used for p-value

### Linear mixed effect model for determinants of personal inhalable paper dust exposure

The linear mixed-effects model on inhalable paper dust showed that the number of machines, the speed of the rewinding machine, and activities conducted in preparation and packing significantly determined the amount of paper dust. Using on average more than four machines in the working rooms increased paper dust levels by 22%. Similarly, the level of dust at the high speed of the rewinding machine was 28% higher than when using such machines at the lower speed. Furthermore, the level of dust increased by 24% and 16% in the preparation and packing job groups, respectively, compared to work in other areas. This finding indicates that the nature of the work being done in different job groups plays a significant role in the level of paper dust present in the air. In the random effect model the the between-worker variance was greater than the within-worker variance. About 29% and 100% of the between-worker and the between-mill variances, respectively were explained by the fixed effects. Totally, about 34% of the variance was explained by the fixed effects (Table 4).

**Table 4:** Linear mixed-effect model of log-transformed personal inhalable paper dust levels among paper mill industry workers in Ethiopia

Fixed Factors	Personal inhalable paper dust			
	Random effects model	Mixed effects model		
	β (SE) *P*-Value	β (SE)	Effect (e^β^)	*P*-value
Intercept	0.53 (0.074) 0.006	0.35 (0.072)		0.003
Average number of machines in the job groups				
≥ 4 machines < 4 machines		0.195 (0.048) Ref.	1.22	< 0.0001
Speed of rewinding machines				
>1900 m/minutes ≤1900 m/minutes		0.246 (0.049) Ref.	1.28	< 0.0001
Job groups				
Packing Preparation Paper machine		0.147 (0.068) 0.217 (0.066) Ref.	1.16 1.24	0.033 0.002
Variance components				
wwδ bwδ bmδ	0.028 (0.0050) 0.041 (0.010) 0.019 (0.018)	0.029 (0.005) 0.029 (0.009) 0.000 (0.000)		
Explained variance by the fixed factors				
Within-worker Between-worker Between-mill		4% 29% 100%		
Total		34%		

β = regression coefficients, SE = standard error of the regression coefficients, wwδ = within-worker variance, bwδ = between-worker variance; bmδ = between mill variance; effect e^β^ = the effect contributed by each determinant; Ref.= Reference

## Discussion

The present study shows that personal inhalable paper dust levels were found to vary across paper mills and job groups. Around 80% of the dust measurements recorded were observed to be higher than the Swedish OEL value of 2 mg/m^3^. The preparation and packing job groups showed a higher level of dust, with an increase of 16% and 24%, respectively, compared to other work areas. The linear mixed model found that the total exposure variability could be attributed to several factors such as job groups, number of machines, and speed of the rewinding machine, which explained 34% of the variability.

Exposure to paper dust varied across paper mills and job groups. Activities conducted in preparation and packing job groups were associated with higher dust exposure compared to work in paper-making machines and finishing and converting. The nature of the tasks involved in the preparation and packing is a significant contributing factor to the increased release of dust particles into the air. The functions in preparation include screening and segregating recycled paper from waste materials, which generates significant dust emissions. Moreover, the handling and movement of paper products, and packing materials using wrappers, labels, or containers, also contribute to the release of dust particles. This is higher than total dust exposure reported in studies conducted in the coupon manufacturing and soft tissue and paper industry in the packing, collection, re-pulping, and storage areas (Fink 2017; Korhonen et al. 2004; Neitzel et al. 2022). However, the level of dust emitted from the paper machine and the finishing and converting job groups during the winding, cutting, and printing of finished products increased dust as compared to other studies (Ericsson et al. 1988; Korhonen et al. 2004). This difference could be due to the raw materials and final product produced in the paper mills being different from the process of soft tissue and coupon manufacturing mills.

The overall result was lower than in a similar study conducted among paper mill workers in Ethiopia (Negash et al. 2023). It is possible that this difference could be attributed to factors, such as two new paper mills added in addition to two old mills studied previously.

In the current study, it has been found that the paper dust level is generally higher compared to personal total dust (Torén et al. 1991) and personal inhalable paper dust (Westberg et al. 2016) found in other paper mill industries. This difference could be attributed to variations in health and safety practices at the workplace and the efficiency of ventilation systems. The level of dust concentration is also higher than in studies conducted in soft tissue paper industries (Neitzel et al. 2022; Sahle et al. 1990; Torén et al. 1991). There could be different reasons for these variations including this study being conducted only in paper mills, whereas the previous studies were in soft tissue mills where the type of products, machine technologies, and working sections varied between mills. However, the current concentration of personal inhalable paper dust was lower than the study done on soft tissue mills (Kraus et al. 2002). The difference may be due to the average sampling time in the previous study being 2 h, while the current study had an average sampling time of around 6 h. A shorter sampling time may have focused on periods during the shift when work activity and exposure are highest.

The mixed-effect model analysis revealed that there is a statistically significant relationship between the number of machines used in a mill room and the level of paper dust emissions. Specifically, paper mills with an average of more than four machines operating in a single room were found to have paper dust emissions that were 22% higher than those with fewer machines. This finding suggests that the number of machines working in a room directly impacts the amount of paper dust generated. In the finishing and converting stage of paper production, rewinding machines are utilized to convert large rolls of paper into smaller rolls or sheets. However, during this process, dust particles can be emitted as a byproduct. Our study found that dust exposure increased by 28% when the rewinding machine is operating at higher speeds compared to lower speeds. This finding also supported that rewinding machines in the paper mills are a significant source of dust emission as also indicated in other studies (Kauppinen et al. 2002; Korhonen et al. 2004). Unfortunately, the production rate per day, which might have explained some of the day-to-day variation in exposure, was not possible to obtain. Also, the type of paper produced could potentially have impacted the amount of dust released from the processes. However, we found it difficult to group the paper mills according to the end product since the majority of the products in the investigated paper mills were similar.

This research study comprehensively analyzed the Ethiopian paper mill industry workers’ exposure to personal inhalable paper dust. The study found that about 80% of the dust measurements exceeded the Swedish OEL (Authority 2018), indicating that measures to reduce the exposure levels should be established. The results of this study provided valuable information that can help develop strategies to reduce the risk of respiratory problems caused by exposure to paper dust by quantifying the amount of paper dust workers are exposed to.

The strengths of this study were that we used repeated measurements in full shift work and an IOM-sampler head was used to measure personal inhalable paper dust exposures. The dust samples were analyzed in an internationally accredited laboratory in Nemko Norway. We used a mixed-effect model to identify the determinants of inhalable paper dust exposure. A limitation of this study was the lack of information such as daily production rate and that ventilation efficiency and housekeeping was not objectively evaluated.

## Conclusions

Employees in the paper mill industry face significant exposure to personal inhalable paper dust, with about 80% exceeding the Swedish OEL. Determinants of exposure were identified including; work in preparation and packing job groups, the use of multiple machines in the working section, and the high speed of rewinding machines. Exposure can be reduced by developing prevention strategies on the technical designing of machines, maintenance of machines, and awareness creation on the safe practice of work.

## Data Availability

The datasets analyzed during the current study are not publicly shared because the datasets contain multiple sensitive identifiers, but the datasets are available from the corresponding author on reasonable request.
